# Understanding molecular consequences of putative drug resistant mutations in *Mycobacterium tuberculosis*

**DOI:** 10.1038/s41598-018-33370-6

**Published:** 2018-10-18

**Authors:** Stephanie Portelli, Jody E. Phelan, David B. Ascher, Taane G. Clark, Nicholas Furnham

**Affiliations:** 1 Department of Biochemistry and Molecular Biology, Bio21 Institute, University of Melbourne, Victoria, 3051 Australia; 2 Department of Pathogen Molecular Biology, London School of Hygiene and Tropical Medicine, Keppel Street, London, WC1E 7HT UK; 3 Department of Infectious Disease Epidemiology, London School of Hygiene and Tropical Medicine, Keppel Street, London, WC1E 7HT UK

## Abstract

Genomic studies of *Mycobacterium tuberculosis* bacteria have revealed loci associated with resistance to anti-tuberculosis drugs. However, the molecular consequences of polymorphism within these candidate loci remain poorly understood. To address this, we have used computational tools to quantify the effects of point mutations conferring resistance to three major anti-tuberculosis drugs, isoniazid (n = 189), rifampicin (n = 201) and D-cycloserine (n = 48), within their primary targets, *katG*, *rpoB*, and *alr*. Notably, mild biophysical effects brought about by high incidence mutations were considered more tolerable, while different structural effects brought about by haplotype combinations reflected differences in their functional importance. Additionally, highly destabilising mutations such as *alr* Y388, highlighted a functional importance of the wildtype residue. Our qualitative analysis enabled us to relate resistance mutations onto a theoretical landscape linking enthalpic changes with phenotype. Such insights will aid the development of new resistance-resistant drugs and, via an integration into predictive tools, in pathogen surveillance.

## Introduction

Tuberculosis disease (TB), caused by bacteria in the *Mycobacterium tuberculosis complex*, continues to have a profound impact on global health. In 2015, TB was responsible for infecting 10.5 million new patients, and taking 1.4 million lives^1^, being the foremost cause of mortality from a single infectious agent. While HIV co-infection and immigration play important roles in disease re-emergence, drug resistance remains an important but poorly understood driver of disease transmission^2,3^, and threatens disease control. Resistance arises from evolutionary selective pressure on *M. tuberculosis* after years of the same-antibiotic re-exposure, patient non-compliance to the long strict drug regime, and a lack of approved and available anti-TB therapies^3^. It is further exacerbated as the latest drugs, such as bedaquiline and delamanid, are reserved as last line add-on drugs^4^ for fear of emerging resistance.

Different *M. tuberculosis* strains have been observed to be both mono- and poly-resistant to current drug regimens, where the latter present a higher risk for treatment failure^5^. Prominent classes of poly-resistant TB strains include: multi-drug resistant TB (MDR-TB), which are resistant to at least isoniazid and rifampicin first line drugs, and extensively drug resistant TB (XDR-TB), which are additionally resistant to at least one fluoroquinolone and one injectable second line drug^1^. Resistance in *M. tuberculosis* may occur genetically or phenotypically, and is often an interplay between the two mechanisms^6^. Phenotypic resistance is a result of changes in gene expression via epigenetic changes and transcriptional regulation, while genetic resistance principally is via acquired genetic mutations, since horizontal gene transfer via plasmids does not occur^5,7^.

Distinctive mechanisms by which resistance is achieved have been described for mycobacteria including *M. tuberculosis*. Intrinsic resistance can manifest in several ways including changes to the cell wall permeability. This can occur through using alternative enzymes involved in cell wall biosynthesis, such as the expression of a second class of transpeptidases that form non-classical linkages between peptides that make up the cell wall leading to resistance in ß-lactam antibiotics^8^. In addition, hydrophilic agents often use porins to cross the outer membrane as the lipid membranes of mycobacteria generally have very low permeability for these agents^9^. The loss of such porins in mycobacteria has shown to drastically increase resistance to hydrophilic antibiotics^10^. As well as low permeability of the cell wall, the other major system contributing to intrinsic resistance are active efflux pumps that transport antibiotics out of the cell. A well-studied exampled in *M. tuberculosis* is the increased transcription of *jefA*, that leads to increased resistance to isoniazid, ethambutol and streptomycin^11^. This increased expression, in clinical strains, is a result of induction in response to antibiotic stress^12^. The importance of efflux pumps has been shown through the development of pump inhibitors that can restore susceptibility in resistant strains^13^. Another intrinsic route to resistance is though modification by acetylation or methylation of either the antibiotic or its target to inhibit interaction or recognition^14^.

A major route to resistance is acquired resistance principally via genetic polymorphism or mutations, including single nucleotide polymorphisms (SNPs), insertions and deletions (indels) and occasionally large deletions in genes coding for drug-targets or -converting enzymes in the *M. tuberculosis* genome (size 4.4 Mbp)^7^. Pharmacologically, these mutations may manifest resistance via different mechanisms, affecting drug activation in the case of prodrugs, its entry to the target via drug permeability, or the drug target itself^7,15^. These genetically acquired mechanisms are influenced by factors such as: the genetic differences between lineages and especially differences in mutation rates^16^; epistatic interactions between mutations within the genetic background of *M. tuberculosis*^17^; and the dynamics within a clonal population that lead to competition within a host between sub-populations possessing emergent resistance associated mutations^14,18^.

An understanding of the molecular consequences of these mutations can provide valuable insight into how resistance develops and the pre-emptive identification of likely resistance mutations. Here we focus on the SNPs associated with resistance to two core first-line drugs, isoniazid and rifampicin, and the second-line drug D-cycloserine. The mechanisms of these drugs are broadly known. Isonazid is a compound that inhibits mycolic acid biosynthesis by binding to an enoyl-acyl carrier protein reductase encoded by the *inhA* gene. It is a pro-drug, which is activated by a catalase-peroxidase enzyme encoded by *katG*^19,20^. Most SNPs causing isoniazid drug resistance are mapped onto the *katG* gene, and lead to a reduction or full inhibition of activity^5^. Rifampicin is a semisynthetic antibiotic that binds to the RNA polymerase β subunit encoded by *rpoB*, inhibiting transcription^21,22^. Most rifampicin resistant mutations are confined to the *rpoB* gene^21,22^, within a 27 residue (81 bp) region known as the Rifampicin Resistance Determining Region (RRDR)^23^. Whereas, D-cycloserine blocks bacterial growth by inhibiting two enzymes involved in D-Alanine metabolism and peptidoglycan biosynthesis, including alanine racemase, which is encoded for by the *alr* gene. It is thought that enzyme overexpression leads to D-cycloserine resistance^7^.

Using a suite of structural bioinformatics tools we sought to evaluate the structural and biophysical effects of putative resistance SNPs in the *alr (*D-cycloserine), *katG (*isoniazid) *rpoB (*rifampicin) genes. In particular, we model the effects of these SNPs within their environments in protein drug targets alanine racemase, catalase peroxidase and RNA Polymerase-β, respectively. Using this approach we successfully identified the molecular mechanisms of known resistance mutations. While we have previously observed correlations between structural features and the resistance they confer^24^, a broader systematic analysis of the structural and biophysical changes leading to resistance will greatly facilitate their integration into genotypic phenotyping programmes. The proteomic structural methods decribed have the ability to inform our understanding of how clinically observed variants lead to resistance, as well as guide the development of compounds less prone to resistance^25–27^. Therefore the proteome-wide scaling up of our approach has the potential to identify new drug targets and forms of resistance.

## Results

### SNPs and protein structures

**Figure 1:**
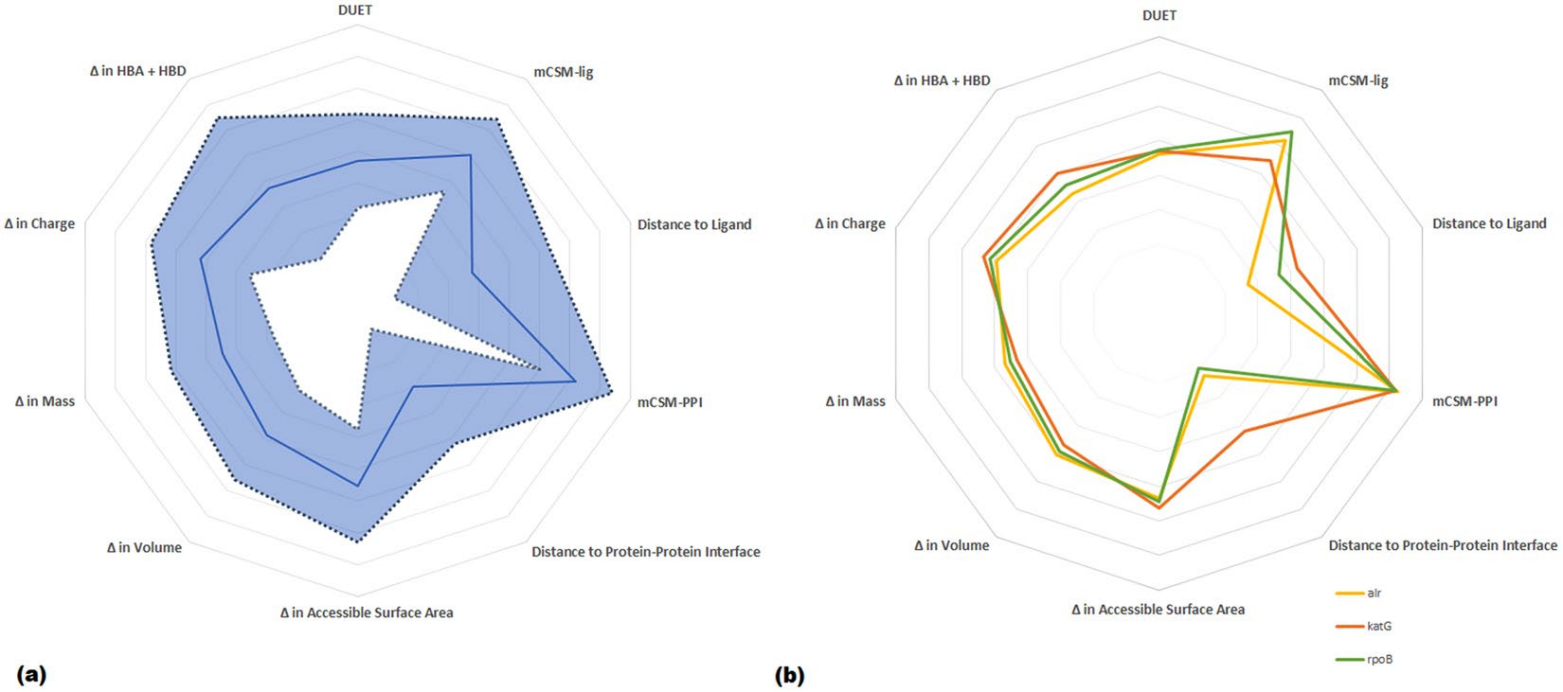
(**a**) General correlations between biophysical (DUET, mCSM-lig, mCSM-PPI) and physicochemical (Accessible Surface Area, Volume, Mass, Charge, Hydrogen bond donors and acceptors) properties of all mutations in this study. The standard deviation for all properties is shown as a shaded area – where the most prominent are changes in hydrogen-bond forming moieties and distances to active site and protein-protein interface. (**b**) Correlations between properties for each target separately: alr in yellow, katG in orange and rpoB in green.

SNP data for 6,465 *M. tuberculosis* clinical isolates with drug susceptibility testing using 1^st^ line and 2^nd^ line drugs were available (described elsewhere)^24^. The isolates represented the four main lineages (1–4) of *M. tuberculosis*, and were sourced from over 20 countries across 5 continents, thereby representing global diversity. Drug resistance phenotypes for the isolates varied from susceptible (n = 4,451, 68.8%), resistant to at least one drug (n = 763, 11.8%), MDR-TB (n = 973, 15.1%) and XDR-TB (n = 278, 4.3%). Many of the isolates have been experimentally measured to establish the minimum inhibitory concentration (MIC) of drug required for resistance phenotype. SNPs with non-synonymous mutations in the *katG* (n = 189) and *rpoB* loci (n = 201) collectively associated with MDR-TB (isoniazid and rifampicin), and within the *alr* gene (n = 48) associated with D-cycloserine resistance were analysed (Supplementary Fig. 1). These SNPs have been identified in recent GWAS studies^24,28^ with the majority (80%) not described in functional studies. The crystal structure of *apo*-alanine racemase is available as PDB entry 1XFC^29^. The crystal structure of *apo-*catalase peroxidase is available as PDB entry 1SJ2, and described elsewhere^19^. The crystal structure of RNA Polymerase-β was inferred through homology modelling. The analytical strategy is described in Supplementary Fig. 2 and the Methods section. In brief, GWAS data and protein structural understandings were used to map SNPs onto protein structures and measure their effects on protein and complex stabilities, and ligand and nucleic acid affinities using the mCSM suites. Based on these measures, a better understanding of molecular mechanisms was achieved based on the predominant functions affected by each mutation. A general overview of all properties and their respective variabilities is depicted in Fig. 1a. As expected: correlation between accessible surface area and volume exists; a weak correlation between mCSM-lig measures and distance from the active site is observed^30^; and other measures showing no correlations to each other (see Supplementary Fig. 3). Trends for the separate proteins are depicted in Fig. 1b, where differences between the three proteins normalised averaged values were intriguingly similar. Notably, the main difference between targets was between affinity and respective distance, and was attributed to the different complexities ranging from small protein Alr to large protein RpoB. Residues having multiple SNPs were identified and linked to drug resistance^31^. These were not limited to the active site, showing that drug resistant hotspots need not directly interfere with ligand binding.

All sites having more than two SNPs were therefore considered as hotspots in our analysis, with sites having only two SNPs could be thought of ‘budding’ resistance hotspots (Supplementary Fig. 1). Within *alr*, four multiple SNP sites having two SNPs each were identified: Thr75, Asp139, Tyr388 and Arg397. The *katG* gene had 22 multiple SNP sites, the most prominent being active site residue Ser315 which had five variants. Its mutation to threonine (S315T) was highly associated with drug resistance^24^, and related to the formation of an isoniazid derivative unable to form INH-NAD^5^. In *rpoB*, 33 sites had multiple SNPs, 13 of which were at the RRDR and within the active site. The most prominent residue of these was His445, also present at the RRDR and active site, for which seven different mutations were identified.

### Most phenotypically resistant mutations acted allosterically

**Figure 2:**
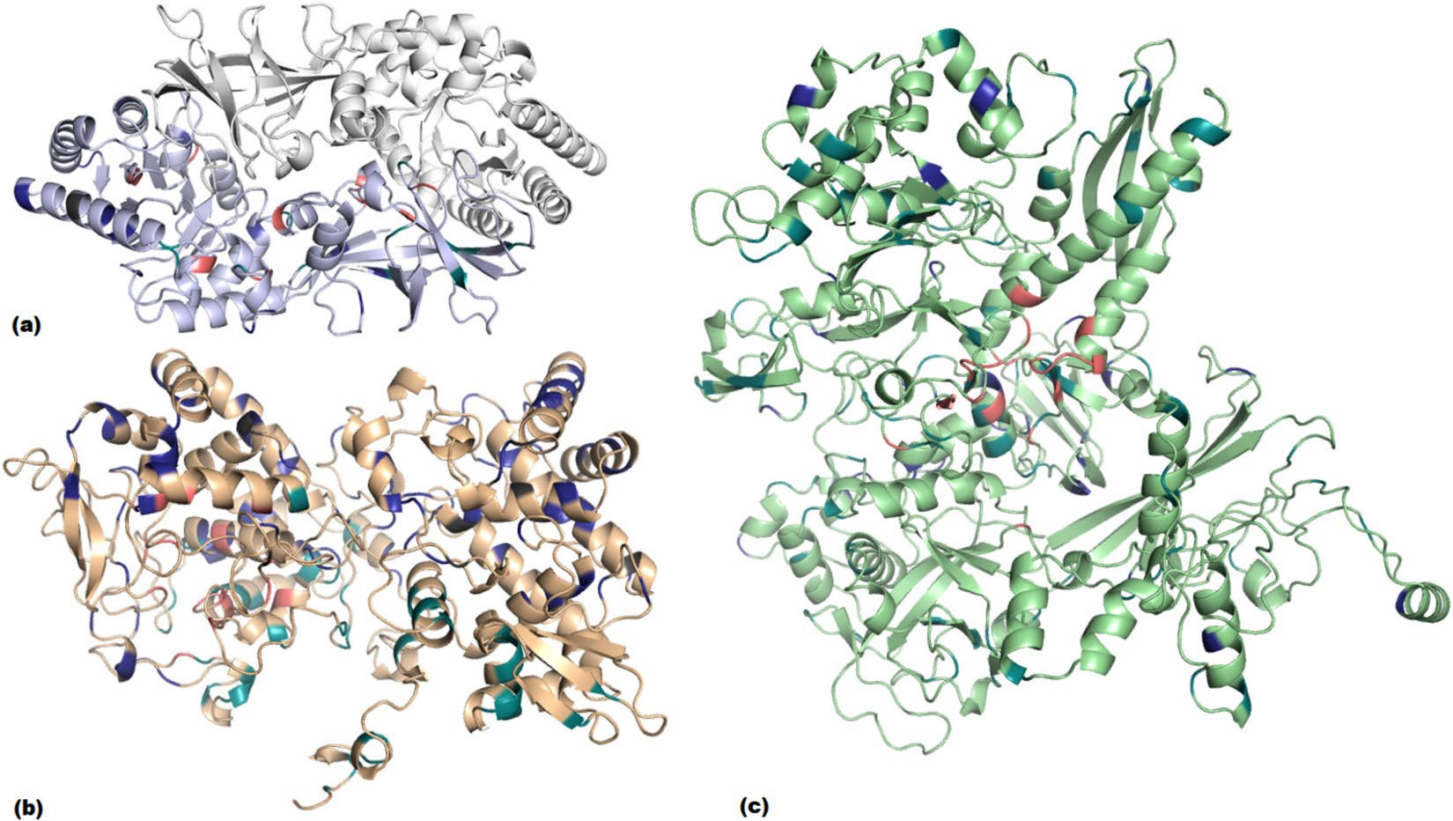
Distribution of mutations within alanine racemase (alr; **a**), catalase peroxidase chain A (katG; **b**) and RNA Polymerase β-subunit (rpoB; **c**). Mutations labelled according to main mechanism: ligand affinity (pink), protein-protein interactions (teal), protomer stability (blue) and unclassified (charcoal grey).

The mutations within our three target proteins were distributed throughout their structures, and were not limited to the active site despite showing the drug resistant phenotype. Only 18% of resistant variants were present within 10 Å of the active site, while the remaining 82% were present at distal sites. Within *alr*, 39.6% of the 48 mutations were within 10 Å of either protomer’s (of the homodimer) active site and 58.3% within 10 Å of a protein-protein interface Supplementary Table 1). The properties analysed were evenly distributed, as 33.3% of all mutations directly affected ligand affinity, 29.2% affected complex stability via protein-protein interactions, and 31.3% were related to protomer stability (Fig. 2). It is thought that this even distribution is due to the small number of mutations identified, the compact structure of the homodimer and the presence of the active site at the protein-protein interface, where different mechanisms imparted by local mutations may equilibrate.

Within *katG*, 14.8% of the 189 mutations were within 10 Å of the active site, while 30.7% were within 10 Å of the protein-protein interface (Supplementary Table 2). The major property affected by variants was protein stability (55.5%), followed by protein-protein interactions as a measure of complex stability (26.5%) and ligand affinity (12.7%; Fig. 2). Since *katG* is not an essential protein for *M. tuberculosis*^32^, but the principle activator of isoniazid, a large proportion of protomer destabilising mutations is thought to impart a drug resistance phenotype through a direct instability of the activating protein, which does not come at a high bacterial fitness cost. For the purposes of our analysis, bacterial fitness in the presence of the drug is considered to be the ability of the *M. tuberculosis* clinical isolates to survive following administration of a drug regimen observed clinically and supported using standard experimentally measured minimum inhibitory concentration (MIC) values, where available.

For target *rpoB*, 29.4% of the 201 mutations analysed were within 10 Å of the active site; 30.3% of all mutations were within 10 Å of any type of nucleic acid – whether single stranded RNA, single stranded DNA, double stranded DNA or DNA/mRNA transcript and 82.6% were within 10 Å of another RNA polymerase complex subunit (Supplementary Table 3). The most prominent properties affected by the mutations were protein-protein interactions (48.3%) with other RNA polymerase subunits, followed by ligand affinity (19.4%), protomer stability (18.4%) and protein-nucleic acid interactions (12.4%; Fig. 2).

Despite the large number of complex destabilising mutations, we know that compensatory mutations are commonly observed within the other subunits^31,33^, which stabilise and retain the function of the full complex. However, as the protein-protein interface comprises both the active site and the nucleic acid binding site, variants affecting local conformation leading to a loss in complex stability, may affect other properties directly leading to drug resistance.

### Mutational effects were mostly imparted via steric or electrostatic changes

**Figure 3:**
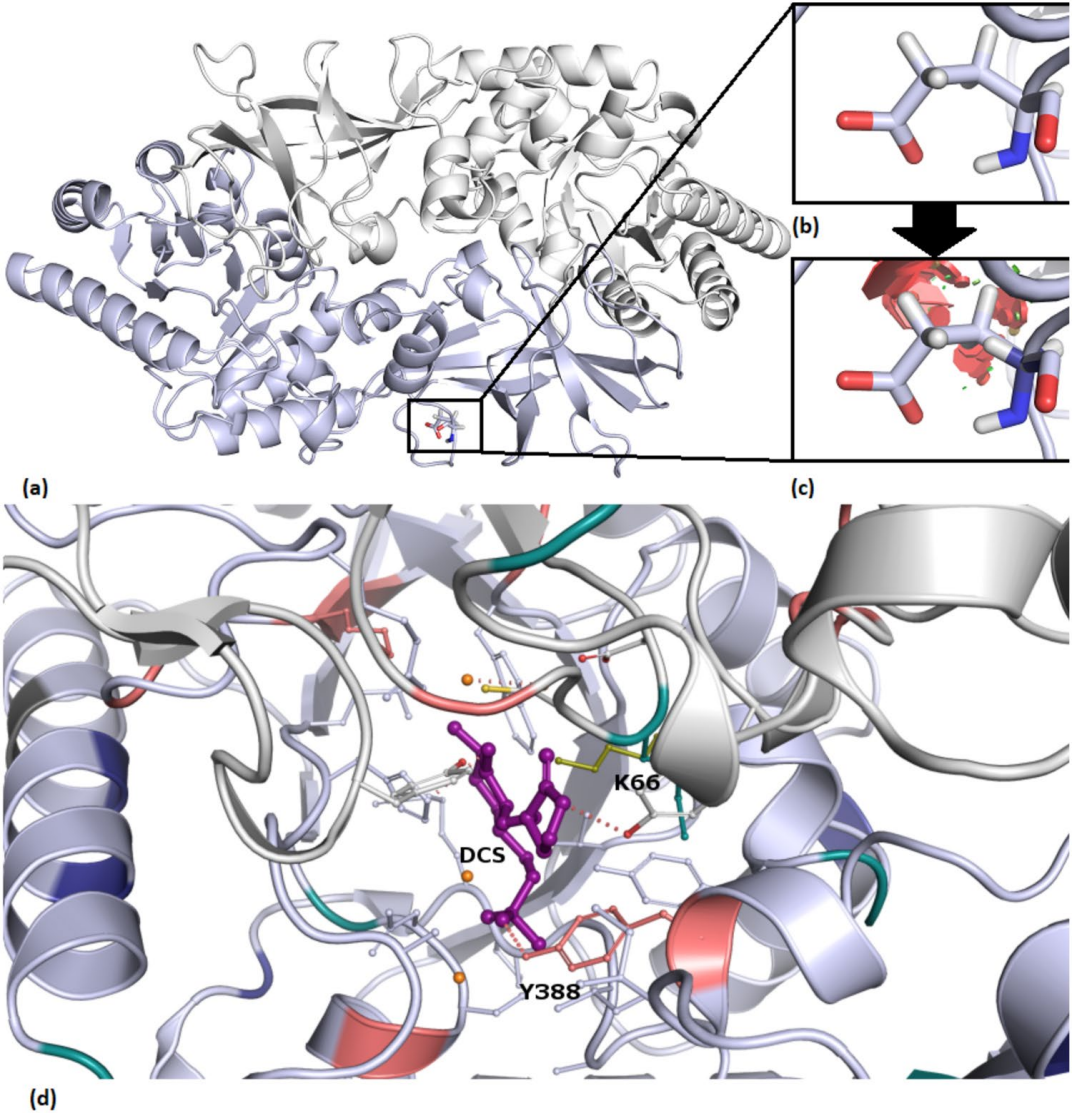
Steric clashes (**c** red) introduced upon mutation in alr when Glu373 (**a**,**b** accessible surface area: 188.42 Å^2^) was mutated to Gly (**c** accessible surface area: 89.41 Å^2^). (**d**) D-Cycloserine(DCS)- pyridoxal 5′-phosphate (PLP) interaction with Y388 at the alanine racemase active site. Lys66, the original residue interacting with PLP, shown in olive green. Mutations labelled according to property: ligand affinity (pink), protein-protein interactions (teal), protomer stability (blue) and unclassified (charcoal grey).

**Figure 4:**
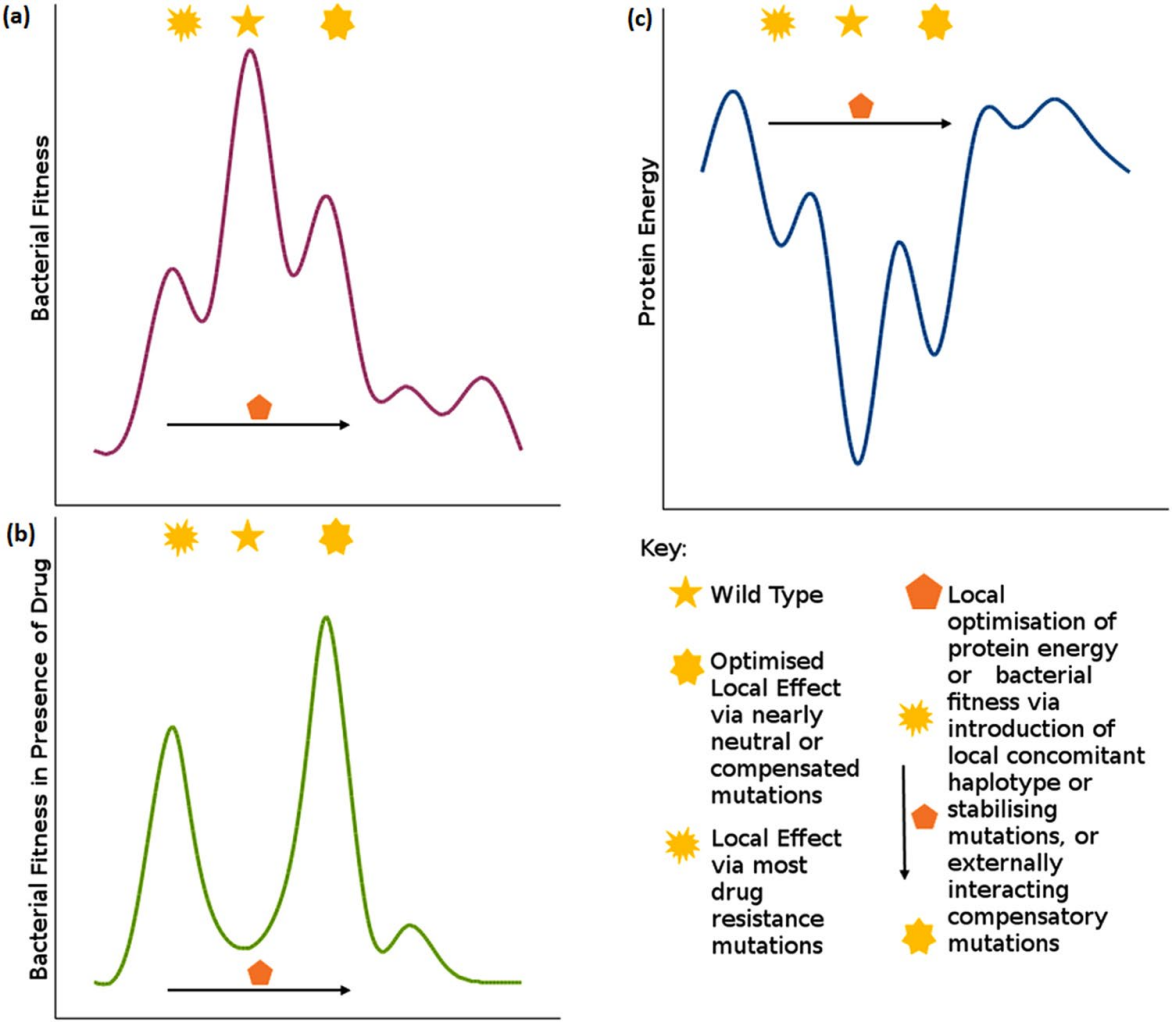
A theoretical bacterial fitness plot in the absence (**a**) and presence (**b**) of drug, running in parallel with a theoretical protein energy plot (**c**) showing different levels of protein minima. Changes in bacterial fitness in the presence of the drug is approximated by the changes clinically observed to susceptibility and supported, where available, by experimentally measured minimum inhibitory concentration (MIC) values from susceptible to degrees of resistance. The ‘Global Minimum’ refers to the overall energy of the protein. This minimum is thought to be higher in the mutant protein when compared to the wildtype, resulting in a lower protein fitness. However, the mutant protein confers a higher cellular fitness to that of the wildtype when subjected to drug selection pressure. (**b**) ‘Local Minimum’ refers to the local changes imparted by low frequency mutations, while ‘Optimised Local Minimum’ is an improved local minimum brought about by high frequency mutations, or concomitant mutations. As an example of values that can attributed to this schematic e.g. katG mutations R463L and S315T: the change in protein energy is −0.215 and −0.154 kcal/mol respectively; the change in bacterial fitness in the presence of the drug is 0.15 and 2.7 respectively as measured by MIC.

At the protein level, local changes in size or charge upon mutation are thought to contribute to conformational changes, leading to a difference in protein stability and interactions with other proteins, nucleic acids and ligands. One such mutation is E373G within *alr* (see Fig. 3). The large steric difference in residue size upon mutation to glycine (accessible surface area: −99 Å^2^), along with a loss of negative charge, has led to a large loss of protein stability (Supplementary Table 1). At the molecular level, this mutation likely disrupts local interactions involving the wildtype residue glutamic acid, causing a local conformational change. Changes in amino acid properties upon mutation were a notable trend throughout the SNPs. Of all the 438 mutations within the three structures, 34.9% exhibited a change in charge, which is judged to affect local interactions with neighboring residues. When considering the change in accessible surface area (ASA; Supplementary Fig. 4) as a measure of residue size, 63.9% of all variants in our dataset exhibited a modulus difference of less than 50 Å^2^. This skewed distribution is considered to be a direct limitation of protein fitness. In the context of protein folding, a large difference in ASA upon mutation is deemed to disrupt local conformation and stability, in turn affecting the overall protein fold. Although we relate this disruption to a protein fitness penalty, other mutations are thought to compensate for these penalties, ultimately restoring protein fitness^32^. However, these conformational changes, though ultimately not affecting overall protein fold, may still lead to functional changes. Therefore, is thought that differences in drug response, coupled with an improved protein fitness via compensatory stabilising mutations, leads to the development of the drug resistance phenotype (Fig. 4).

### Frequently occurring mutations did not confer extreme changes in parameters

The highest incidence GWAS mutations within alanine racemase (L113R, Supplementary Table 1) catalase peroxidase (R463L and S315T, Supplementary Table 2) and RNA polymerase-β (S450L, Supplementary Table 3) conferred relatively mild changes to the effects measured when considered individually. When considering these effects in relation to protein fitness, these mutations are deemed to be nearly neutral, due to a lower effect on protein stability. This lower effect leads to a lower protein fitness penalty, which is thought to lead to their enrichment within a population. Therefore, within an energy landscape (Fig. 4c) these mutations are considered to reside at an ‘optimised local minimum’, closer to the wild type global minimum. Due to this lower protein fitness penalty, these mutations are also thought to confer a good fitness at the bacterial fitness level, with an improved bacterial fitness in the presence of drug, due to their drug resistance phenotype.

The *katG* mutation R463L had mild to moderate destabilising (therefore showing a reduction in free energy values in the measures tested) effects despite its high incidence (40.6%), however it showed a lower association with drug resistance (*p-*value: 6.75 × 10^−1^) than S315T (*p*-value: 6.60 × 10^−1^)^34^ because R463L is a lineage-specific mutation, which was controlled for in the regression-based analysis. While R463L seems to have a higher fitness penalty than expected for its frequency, this mutation is part of a number of haplotype combinations (described later in further detail). It is therefore postulated that these secondary mutations that combine with R463L compensate for this fitness penalty, and enable the enrichment of the mutation within the bacterial population (Fig. 4). The *alr* high frequency mutation L113R is another exception to the nearly neutral concept, as it showed highly destabilising effects on both ligand affinity and protein stability. These values may explain why the incidence of this mutation, though the highest in its subset (0.57%), are very low compared to other high incidence mutations (R463L: 40.6%, S315T: 18.5%, S450L: 13.5%). Both exceptions (R463L in *katG* and L113R in *alr*) are associated with a change in electrostatic properties, considered to disrupt the local conformation and thereby effect protein folding.

On average, the effect of these four high incidence mutations on protein stability was mildly destabilising (DUET average: −0.43 kcal/mol; DUET median: −0.285 kcal/mol), while that of lower incidence mutations was highly destabilising (DUET average: −1.01 kcal/mol; DUET median: −0.939 kcal/mol). When considering steric changes for these high incidence variants, only *rpoB* mutation S450L had an ASA difference larger than 50 Å^2^. While this was outside the majority of cases, a large change in ASA (78 Å^2^) for this residue could be attributable to compensatory mutations occurring in RNA polymerase-β‘ subunit (*rpoC*)^33^, which allow for steric difference. Notably, the *rpoC* mutation V483G (modulus difference in ASA: 80 Å^2^) has been identified as a compensatory mutation for S450L^33^, which cancels out the change in ASA. In turn, this is considered to optimise the local energy minimum at this residue locus (Fig. 4c), thereby increasing bacterial fitness.

Finally, conservation scores from ConSurf^34^ for the frequent mutations showed that L113R (−0.925; *alr*), S315T (−0.834; *katG*) and S450L (−0.771; *rpoB*) had a slow evolution rate (denoted by a negative value), and were therefore highly conserved. On the other hand, R463L (0.225; *katG*) had a fast evolution rate (denoted by a positive value), showing a less conserved site. We relate these scores to the higher destabilising effects exhibited by R463L when compared to its high frequency counterparts, and a lower association with INH resistance when compared to S315T. We consider high changes (>±1.00 kcal/mol) in mCSM^30,35^ and DUET^36^ predicted ∆∆G to be unlikely to be stable enough to be passed on from one generation to another, resulting in a lower population incidence. Moreover, we attribute large steric (>50 Å^2^) changes to a local instability, that is unlikely to be tolerated without external, compensatory mutations. The conservation scores further highlight our observations, as the mutations at these highly conserved residues were also related to drug resistance in GWAS (L113R *p-*value: 6.44 × 10^−17^, S450L *p-*value: 2.85 × 10^−10^ ^2^). More tolerated mutations are retained within the population via an evolutionary selective pressure - drug exposure, and therefore results in the high frequency mutations within a GWAS analysis^24^. While the effect of drug resistant mutations on bacterial fitness and virulence is still debatable^37^, we relate the above tolerable ∆∆G values of frequent mutations to a low fitness penalty for *M. tuberculosis* strain survival (Fig. 4).

### Mildly stabilising mutations may confer local fitness advantages

We have classified our protein variant subsets depending on the most prominent destabilising effect imparted, considering ligand affinity, nucleic acid affinity, complex stability (as protein-protein interactions) and protomer stability in a sequential order. As a result, a minority of mutations within each subset remained residual (denoted as ‘unclassified’), as they did not confer negative enthalpic effects, but mild overall positive effects. Similarly to the high frequency mutations described above, unclassified mutations are also considered to be nearly neutral, but are thought to have an opposite role via a mildly stabilising effect. In general, all unclassified mutations mapped within a 5 Å distance to destabilising mutations and described in more detail below. Despite a lack of GWAS evidence showing haplotype combinations including these unclassified mutations, we associate the overall positive effects to a compensatory protein fitness measure (Fig. 4) for neighbouring destabilising mutations. In turn, this enables compensatory evolution, enriching resistant mutations within a bacterial population, as previously described^38^.

Three *alr* mutations (T75M, A217V and T401I; Supplementary Table 1) remained unclassified as per our analysis. All three mutations were present at the solvent accessible region of the protein, and far away (>10 Å) from the interacting regions. Notably, mutation A217V was close to (<5 Å) A212D and F215V, which were highly destabilising for protomer stability. Theoretically, it is thought that A217V improves the fitness of these close mutations by improving a local minimum (Fig. 4c), however, there was no GWAS evidence of these mutations occurring concomitantly. A similar scenario is applicable to T401I, which is close to variant A38V, which is moderately destabilising for protomer stability. Mutation T75M was not present close to any other mutation in our dataset, but was at a polymorphic site also mutated to alanine. As this residue was not within an interacting distance to the drug or the interface, a possible mechanism of resistance is a compensatory action against unfavourable closeby mutations thereby maintaining a local protein minimum (Fig. 4c). We relate an improvement in local minima to an increased protein and bacterial fitness, especially in the presence of the drug (Fig. 4b).

Within *katG*, 5.8% (S211N, D259Y, E452Q, T475I, S481L, D511N, E523K, Q525P, K557N, L598R and G630V) of mutations remained unclassified according to our analysis. All mutations are present at or close to the solvent accessible region and within 5 Å of highly destabilising mutations (Supplementary Table 2). It is thought that these unexplained favorable effects imparted via these mutations may cancel out neighbouring disruptive mutations, reducing their fitness penalty to a more tolerable energy minimum (Fig. 4c). Notably, within this protein, most of the unexplained mutations (except S211N and D259Y) are present at the C-terminus, surrounded by predominantly protomer destabilising mutations. Hence, the protomer stabilising nature of these unclassified mutations may also help restore the local protein fold, increasing protein fitness and overall bacterial fitness.

Only 1.5% of *rpoB* mutations (E66K, D259N and T676A; Supplementary Table 3) remained unclassified following our analysis. E66K is present within the solvent accessible surface, within 5 Å of variant A69P which disrupts complex stability. D259N is also at the solvent accessible surface, with no other mutations within 5 Å. T676A is present within 10 Å of rifampicin binding, and within 5 Å of mutations S672Y and G675D that are highly destabilising for protomer stability, along with H1028R, which affects ligand affinity. The mildly stabilising effect of T676A may help to cancel out local protein instability at this region and maintain a local energy minimum (Fig. 4c), leading to the enrichment of the drug resistant mutations within the bacterial population, and enabling bacterial fitness in the presence of rifampicin (Fig. 4b).

### Haplotype combinations exhibit different means of achieving resistance concomitantly

The role of secondary concomitant mutations have been previously described as having a stabilising role within a protein structure, in order to restore a fitness penalty imparted by highly destabilising mutations^39–41^. Within our fitness plot (Fig. 4) these concomitant haplotype mutations are thought to improve the local protein energy minimum, leading to the improvement of the bacterial population fitness, particularly in the presence of the drug.

**Figure 5:**
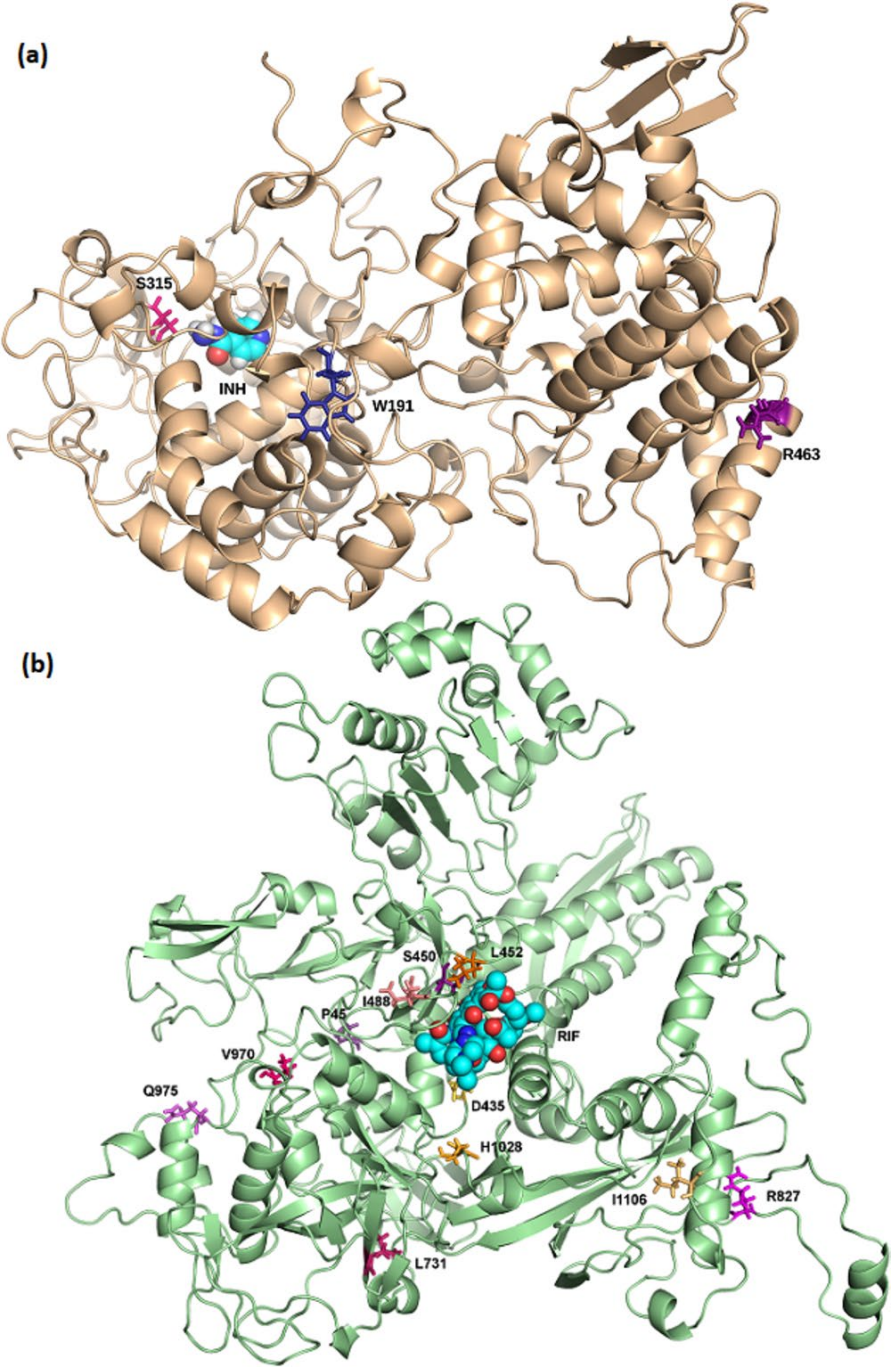
(**a**) katG haplotype combinations between R463 and W191/Ser315 residues. (**b**) Haplotype combinations occurring in rpoB. Those coupled to S450 are depicted in the purple colour scale, while those coupled to L452 are rendered in the orange colour scale. Mutated residues are shown as sticks, while isoniazid (INH) and rifampicin (RIF) are shown as spheres in cyan.

Three haplotype combinations were identified within the *katG* gene (Supplementary Table 4), where the most prominent combined effects were a reduction in INH affinity and complex stability. Mutation R463L, located at the solvent accessible C-terminus, was present in all haplotypes. Its combination with W191R at the N-terminal protein-protein interface, and S315T/N at the active site is thought to be a means of achieving different resistance mechanisms concomitantly (Fig. 5a). Moreover, W191R and S315T/N are thought to compensate the effects of R463L, and enrich it within the population. On the other hand, the eight *rpoB* haplotypes (Supplementary Table 5) identified were more dispersed, and included residues having multiple SNPs: S450, V970, R827, P45 and D435 (Fig. 5b). Most haplotypes occurred coupled to S450L, the most frequently occurring mutation for this protein. Again, it is thought that these haplotypes may help enrich S450L within the population by counteracting any steric and consequentual conformational changes imparted via S450L (change in ASA: 78 Å^2^). Mutations coupled to L452P were present both at the active site (D435G, Fig. 5a), and at the β′ interface (I1106T and H1028R, Fig. 5b). L452P combinations were moderately destabilising for protomer stability, ligand affinity and complex affinity, while they were mild to moderately stabilising for nucleic acid affinity. From those coupled with S450L, only I488V is in close geographical proximity to the active site (Fig. 5b), which explains the highly destabilising effect on nucleic acid affinity occurring for this combination. Q975H and V998A are at the α1 interface, L731P lies at the interface between all four subunits, and R827C is close to the DNA binding locus (Fig. 5b). From these observations, we conclude that a close topological proximity of concurrent mutations is thought to enable localised effects, while the combination with external mutations ensures different mechanisms lead to drug resistance concurrently.

### The prominent *alr* Tyr388 mutation

Certain loci within our targets are thought to be at strategic positions, as reflected in their spectrum of effects. The most pronounced example of a strategic locus in our dataset was residue Y388 within alanine racemase. This residue was confirmed by our ConSurf^34^ analysis to be highly conserved, indicating a potential functional significance for *alr*. Properties for drug resistance, as discussed above, are exemplified in Y388. Multiple mutations at the site (Y388D/C) show linkage to a developing mutational hotspot for drug resistance^31^. Both SNPs at this locus are the most prominently disruptive within alanine racemase (Supplementary Table 1), exhibiting destabilising effects across all the enthalpic measurements taken. Residue Y388 is situated within the active site entryway and forms a 2.7 Å gate with Y295 of the opposite chain^29^. The smaller aspartic acid and cysteine mutant residues are thought to destabilize this gate, via a loss of hydrophobic inter-tyrosine interactions and subsequently, protein-protein affinity. The loss of the van der Waals interaction between Y388 and PLP (Fig. 3d), in turn, is thought to reduce ligand stability, possibly affecting affinity.

## Discussion

Genetic variants, including nsSNPs, present within drug targets and drug-activating enzymes have been identified as a main cause of drug resistance in TB^7^. In this study, we aimed to understand the consequences of nsSNPs, to better understand driving forces of drug resistance in TB. We have related mutations identified from clinical isolates to local molecular mechanisms, using *in-silico* measures of change in enthalpy. The mCSM^30,35^ predictions use graph based signatures, where inter-atomic distance patterns describe the wild-type residue environment directly interacting with the mutation site. Long range patterns are also considered^30,35^, meaning that both local and global effects of point mutations have been characterized. Appended to these signatures within mCSM is a ‘pharmacophore count’ vector, which considers the atom changes from wildtype upon mutation^30,35^. These features are present within all mCSM^30,35^ tools used, while DUET^36^ uses a consensus regression score of predictors mCSM-Stability^35^ and SDM^42,43^. The latter method calculates amino acid frequency substitutions within homologs to reach a stability score^42,43^. Therefore, DUET^36^ is thought to be a better predictor for protein stability upon mutation than mCSM^35^ alone. Through these methods, we identified how our point mutations affected protein stability and protein interactions with other proteins, ligands or nucleic acid^44,45^, and subsequently how these changes affected protein function^44,46^.

Our study focused on categorising the predominant effect of each mutation, based on distance to interacting site. The destabilising effects were given higher consideration in classifying mutations, since nsSNPs are usually related to a decrease in protein stability, or a change in protein function^41^ (via disruption of drug affinity, nucleic acid affinity or overall complex stability). This left a minority of protein stabilising variants ‘unclassified’, which we consider to be nearly neutral for protein fitness. Their stabilising role is thought to permit the restoration of the protein fold following the introduction of conformation-disrupting, unfavourable mutations in close proximity (Fig. 4c). The restoration of the protein fold is thought to increase the bacterial fitness in the presence of the drug (as approximated by changes in clinically observed susceptibility supported by minimum inhibitory concentration (MIC) values), as drug resistant mutations are stabilized and enriched within the population^41^ (Fig. 4a,b). Moreover, an increase in protein stability has been previously linked to a detrimental effect on protein dynamics and regulation^47^, which further supports their role in conferring local stability and protein fitness (Fig. 4c), despite not being present in any of our haplotype combinations. Since all were close to highly destabilising mutations, a stabilised protein conformation enables these highly unfavourable mutations to become tolerated, suggesting a possible mechanistic approach to achieve drug resistance.

The overall protein fitness is thought to be dependent on the extent of enthalpic measures, concomitant mutations, and compensatory mutations in interacting proteins (Fig. 4c). The milder effects on protein stability exhibited by the high frequency mutations are considered to help select for the mutation enrichment within the population. High frequency mutations, along with the unclassified mutations, are deemed to be nearly neutral with respect to protein fitness. The ‘unclassified’ variants had mild stabilising effects, while high frequency mutations had mild destabilising effects. This enabled the latter to confer drug resistance via an optimised local energy minimum (Fig. 4c), thereby considered to be better tolerated according to protein thermodynamics. Further to this, the nearly neutral nature of high frequency mutations is thought to enable their enrichment in a particular locus, due to an overall ‘mild impact’ imparted on protein folding and bacterial fitness, whether in the presence or absence of drug (Fig. 4). The more destabilising mutations are considered to confer a ‘higher impact’ on protein folding energy, and subsequent protein and bacterial fitness, and are deemed to be less likely tolerated. Within the energy fitness curve (Fig. 4) these mutations lie at the higher protein energy (Fig. 4c) and a lower bacterial fitness (Fig. 4a,b), but are considered to be stabilized by compensatory effects to an optimised protein energy minimum, leading to a higher bacterial fitness (Fig. 4a,b). These compensatory effects are thought to occur via concomitant mutations, either within interacting proteins e.g. RNA polymerase subunits^33^, or via same-protein haplotype combinations. Some mutations affected different properties measured due to their distances from the interacting surfaces. These measures are considered to collectively help enrich a mutation and bring about the drug resistance phenotype.

Trends in variant distribution within all three proteins studied have shown that most GWAS phenotypically resistant mutations were not present at the active site, meaning that the effect was brought about allosterically, not directly through ligand binding affinity. Allosteric effects are predominantly via confiring protomer stability for *alr* and *katG*, and non-ligand interaction affinities for *rpoB*. While we do not have the MIC values for all the SNPs to relate *in-silico* results to experimental meaures of resistance, this was the most in-depth study identifying enthalpic effects of SNPs within *M. tuberculosis* targets catalase peroxidase, RNA Polymerase-β and alanine racemase to date. Through this qualitative analysis, we have observed what are considered to be fitness penalty measures imparted through the point mutations, where the same variant exhibits drastic enthalpic costs in one of the properties tested, but is compensated with balancing, favourable effects on other measures (Supplementary Tables 1–3). Further, haplotype sets have shown concomitant, complimentary effects, possibly to promote fitness of strain survival (Fig. 4). These same haplotype combinations are thought to purposely occur near to each other to enhance a local mechanistic effect, or at separate parts of the target to enable multiple mechanisms of resistance concomitantly.

Our analysis has shed light on new possible SNP hotspot Y388 within alanine racemase, which confers highly destabilising effects on general protein stability, and affinity for homodimer formation and D-cycloserine ligand binding. Within this residue, a disruption of local electrostatic bonding is thought to occur in both variants, via an addition of a negative charge (Y388D) or an addition of a sulphur atom (Y388C). However, the mutation to aspartic acid was more disruptive than that to cysteine, potentially due to the introduction of a residue having negative charge.

The improved understanding of mechanistic effects of drug resistant TB strains is of ultimate importance for the design of newer, resistance-resistant therapies, via the avoidance of drug resistance hotspots, and the development of machine learning approaches to predict resistance potential of SNPs. This is crucial in a disease state which has seen only recent introduction of newer therapies^4^, where poly-resistant strains have had time to develop, and affects millions of people worldwide every year. An understanding of mutational structural mechanisms can be applied to other infectious and non-infectious diseases under selective drug pressure, since the principles of protein structure and function are universally applicable.

## Materials and Methods

### Drug Targets and the SNP dataset

SNP data and association *p*-values were available from a GWAS of 6,465 clinical isolates, which had drug susceptibility test data for rifampicin, isoniazid and D-cycloserine^24^. Simultaneously we compiled a list of all the anti-TB drugs and their respective targets from the literature, and a search for suitable crystallographic structures was initially made in PDBe^48^ and RCSB^49,50^ databases. CHOPIN^51^ and ModBase^52^ homology databases were used to identify homology models where crystallographic structures were unavailable. The complete list was ranked according to SNP *p*-values from the GWAS. Due to their strong associations with MDR-TB^1,5,7^ genes *katG* and *rpoB*, which transcribe catalase peroxidase and RNA polymerase-β respectively were chosen, while gene *alr*, which transcribes alanine racemase, was selected due to lack of prior investigation. Within the *katG* gene, 6,733 clinical isolates were tested for isoniazid and 189 nsSNPs, along with 3 haplotype combinations were identified. Within the *rpoB* gene, 6,697 isolates were tested for rifampicin, which identified 201 nsSNPs and 8 haplotype combinations. In the case of *alr*, 252 clinical isolates were tested for D-cycloserine (DCS) susceptibility, which identified 48 nsSNPs and no haplotype combinations. These nsSNPs were subjected to analysis according to the workflow in Supplementary Fig. 2.

### Protein Modelling

Missing residues from the *alr* crystal structure (PDB ID: 1XFC)^29^ were modelled using UCSF Chimera 1.11^53^ from the second chain in the homodimer. Both chains were kept for docking, as were co-crystallized water molecules present within the active site. Protein minimisation was not possible due to the presence of covalent linkage between residue Lys66 and PLP. Instead, Molprobity^54,55^ was used to assess protein suitability due to modelled residues, while WinCoot-0.8.3^56^ was used to ‘regularise’ outlying residues identified within the structure. Prior to docking DCS-PLP, the PLP bonded to Lys66 was removed using UCSF Chimera 1.11^53^.

Proteins *alr* and *katG* were present as previously described crystallographic structures, while the homology model of rpoB was built on templates PDB IDs: 1TWF^57^, 1YNN^22^ (holoprotein), 2A6H^58^, 2E2I^59^ and 3CQZ^60^ using Schrondinger Suites v.2016.4.

### Homology modelling: RNAP complex

For use in the mCSM-PPI and mCSM-NA^35^ predictions, the full RNA polymerase complex was required. At the time of the study, the now crystallised *M. tuberculosis* structure (PDB ID: 5UHC)^61^ was not available, therefore a homology model was used instead. The homology model of the full *M. tuberculosis* RNAP was built using the crystallised structure of *E. coli* RNAP^62^ as a template. Homology models of the α1, α2, and β‘ *M. tuberculosis* subunits were initially searched for in CHOPIN^51^ and Phyre2^63^, using the amino acid sequences available in Tuberculist^64,65^ as search queries. Models for α1 and α2 were obtained from Phyre2^63^ using template PDB ID: 5I2D, while the β‘ model was obtained from Phyre2^63^ using PDB ID 4G7O as the template. The ω subunit was not modelled as it did not directly interact with the β subunit, and therefore was outside the scope of our model. The individual subunits were aligned to the *E. coli* subunits^62^ and the co-crystallised DNA from the *E. coli* structure was modelled onto the *M. tuberculosis* structure^62^, using UCSF Chimera 1.11^53^. Upon the availability of the crystal structure PDB ID 5UHC, our modelled complex was superimposed with the crystallised complex using PyMol^24^. The two structures differed in backbone atomic positions at a root-mean square deviation (RMSD) value of 2.38 Å over 6596 atoms. Given the 3.8 Å resolution of the crystal, these RMSD values give us reasonable confidence to assume our model is of good quality as its atomic positions are within the margin of error of the crystal structure obtained.

### Ligand Docking

The apo-protein structures of *alr*, *katG* and *rpoB* required docking of their respective ligands for further analysis using mCSM-Lig^30^. The ligand structures for DCS (covalently bound to pyridoxal 5′-phosphate (PLP); DCS-PLP), isoniazid and rifampicin were obtained from *holo*-homolog structures PDB IDs 4LUT^66^, 5SYJ^20^ and 1YNN^22^ respectively. The first and second line drugs DCS (as DCS-PLP), isoniazid and rifampicin were docked within *alr*, *katG* and *rpoB* structures respectively, using Glide (Schrodinger Suite). Docking was carried out using default parameters, guided by the positions of homolog co-crystallised ligands in PDB entries 4LUT^66^ (*alr*), 5SYJ^20^ (*katG*) and 1YNN^22^ (*rpoB*). To facilitate analysis, DCS-PLP was only docked within chain A of *alr*, although both chains were kept for docking. While cofactor heme was kept for isoniazid docking within *katG*, cofactor PLP was docked covalently bound to DCS within *alr*. The best binding poses were chosen through an RMSD comparison with the homolog-bound ligands, and their interaction with active site residues through an Arpeggio^67^ analysis.

### *In silico* predictions

The GWAS SNP hits were subjected to the graph-based signature algorithms: DUET^36^, mCSM-Stability^35^, mCSM-Lig^30^, mCSM-PPI^35^ and mCSM-NA^35,68^ (where applicable). Data was filtered according to distance from interacting site (ligand pose, protein-protein interface and nucleic acid interface), where residues at a distance >10 Å were excluded. These were classified according to ∆∆G numerical thresholds: highly destabilising (<−1.00 kcal/mol), moderately destabilising (between −1.00 and −0.5 kcal/mol), mildly destabilising (between −0.5 and 0 kcal/mol), mildly stabilising (between 0 and 0.5 kcal/mol), moderately stabilising (between 0.5 and 1.00 kcal/mol) and highly stabilising (>1.00 kcal/mol). Finally, distances to the interacting sites were also categorised in two: <5 Å (likely interacting) and <10 Å (possibly interacting). Conclusions were then drawn up based on all the factors and interaction distances. In classifying the major properties of each mutation, the destabilising effects were reviewed in a hierarchical manner, according to ligand affinity, nucleic acid affinity, complex stability (protein-protein interactions) and protomer stability. Unexplained mutations were those which had a stabilising effect, with no other destabilising effects to explain disruption of the wildtype.

### Haplotype analysis

During haplotype analysis, we sought to calculate the enthalpic change in properties upon introduction of a mutation, in the presence of another mutation. Mutant proteins representing single mutations were initially prepared using PyMol^24^ mutagenesis wizard. These were analysed using the tools described above. The values obtained from each haplotype variant were then averaged with the other variants in the same combination, in order to obtain a consensus value depicting overall effects. To calculate the value for each property (*y)* and haplotype mutations A and B occurring concomitantly, we used the equation

$${y}_{haplotype}=\frac{({y}_{Am\_B}+{y}_{Bm\_A})}{2},$$

where *Am_B* refers to the protein mutated with variant *A*, followed by introduction of mutation *B* within the mCSM webtools*; Bm_A* refers to the protein mutated with variant *B*, followed by introduction of mutation *A* within the *mCSM* webtools.

## Electronic supplementary material

Supplementary Information Figures

Supplementary Table 1

Supplementary Table 2

Supplementary Table 3

Supplementary Table 4

Supplementary Table 5
