# Supplementary material for: Understanding molecular consequences of putative drug resistant mutations in Mycobacterium tuberculosis
Source: Sci Rep. 2018 Oct 18;8:15356. doi: 10.1038/s41598-018-33370-6 (PMC6193939; doi:10.1038/s41598-018-33370-6)
Supplement: Supplementary file 1 — Supplementary Information Figures [file 41598_2018_33370_MOESM1_ESM.pdf]

Supplementary Information

Title:

Understanding molecular consequences of putative drug resistant mutations in *Mycobacterium tuberculosis*

Authors:

Stephanie Portelli[1], Jody E. Phelan[2], David B. Ascher[1], Taane G. Clark[2,3,*] and Nicholas Furnham[2,*]

1. Department of Biochemistry and Molecular Biology, Bio21 Institute, University of Melbourne, Victoria, Australia, 3051

2. Department of Pathogen Molecular Biology, London School of Hygiene and Tropical Medicine, Keppel Street, London, WC1E 7HT

3. Department of Infectious Disease Epidemiology, London School of Hygiene and Tropical Medicine, Keppel Street, London, WC1E 7HT

* Joint authors

Corresponding author:

Dr. Nicholas Furnham

Department of Pathogen Molecular Biology

London School of Hygiene and Tropical Medicine

Keppel Street, London WC1E 7HT

Nick.Furnham@lshtm.ac.uk

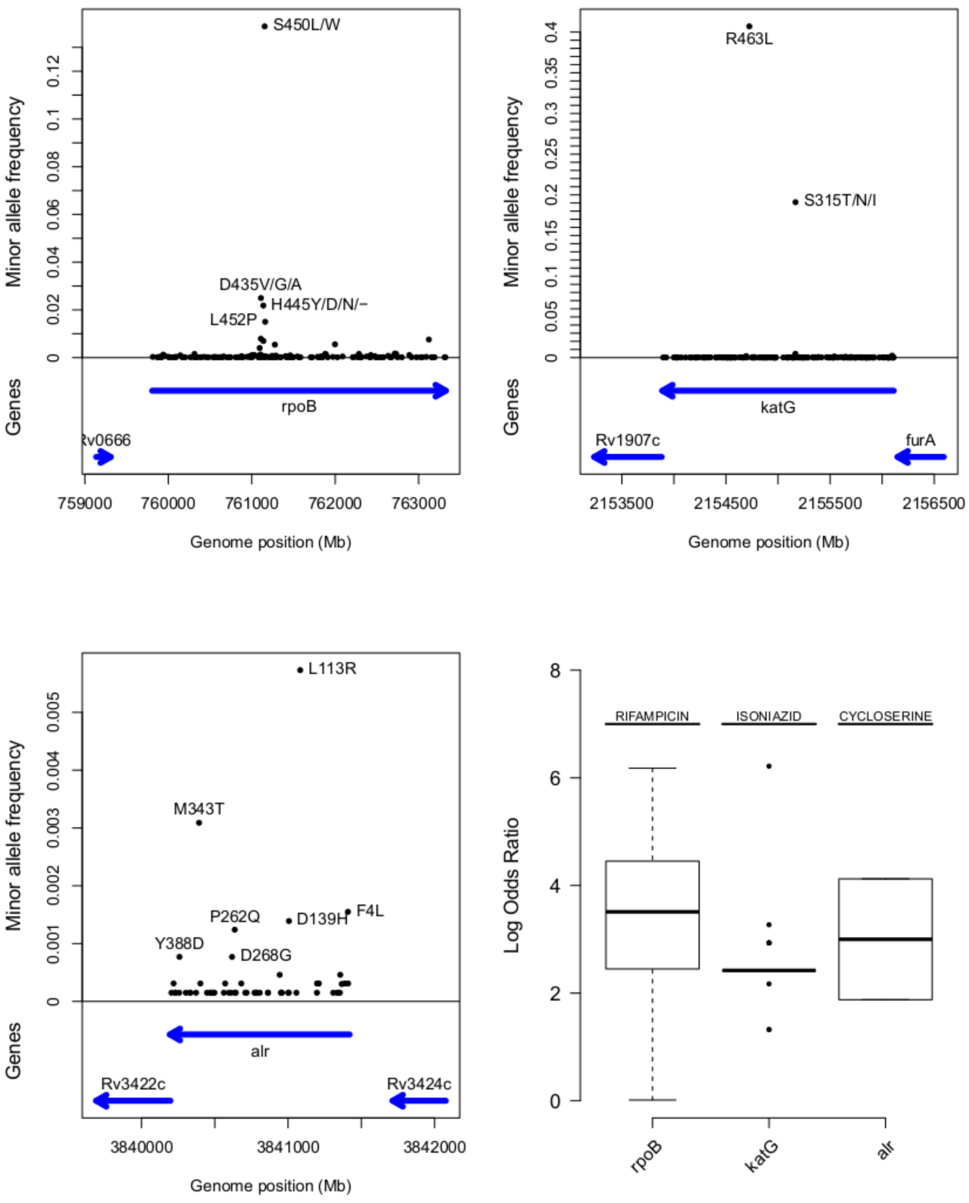

Supplementary Figure 1: Gene maps showing allele frequencies, and histograms of multiple SNPs as obtained from GWAS. SNPs included in this study were those which mapped onto the available protein structures.

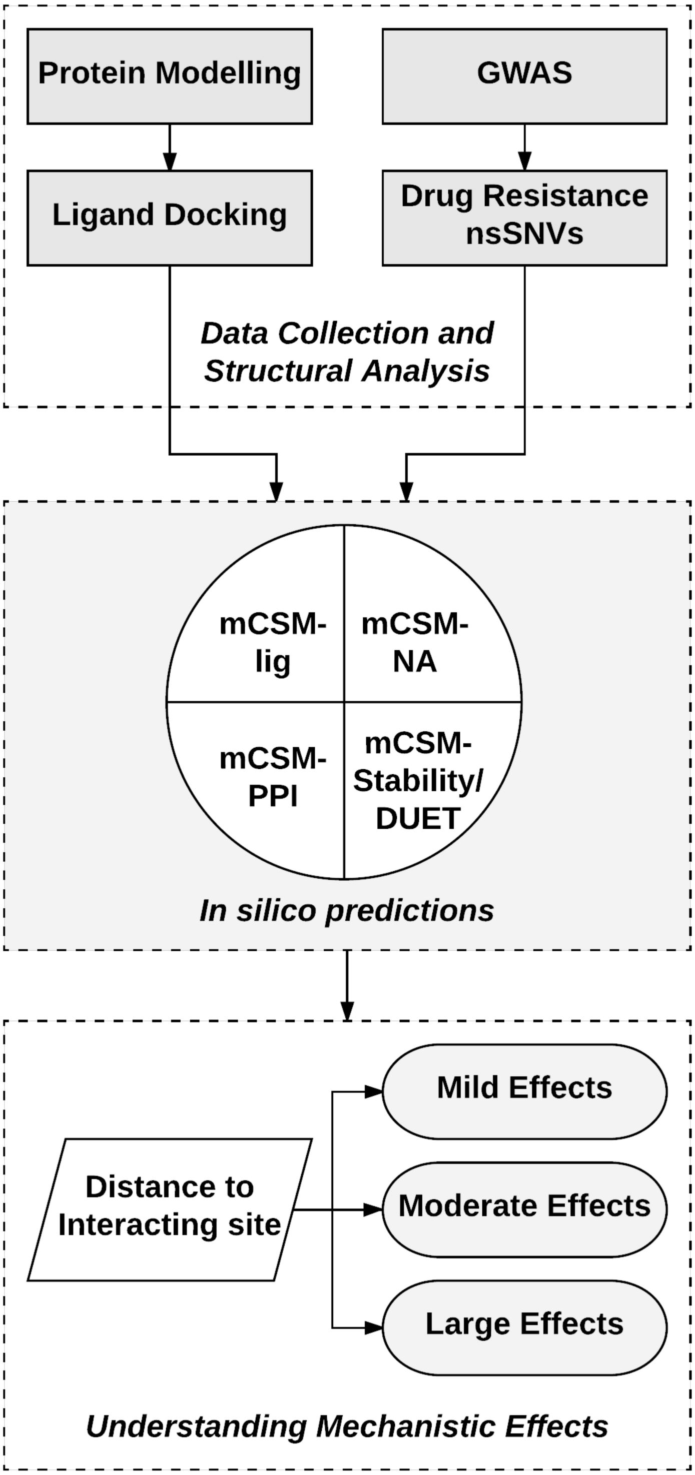

Supplementary Figure 2: General workflow adopted in this study. GWAS: Genome Wide Association Study, nsSNVs: non-synonymous SNPs.

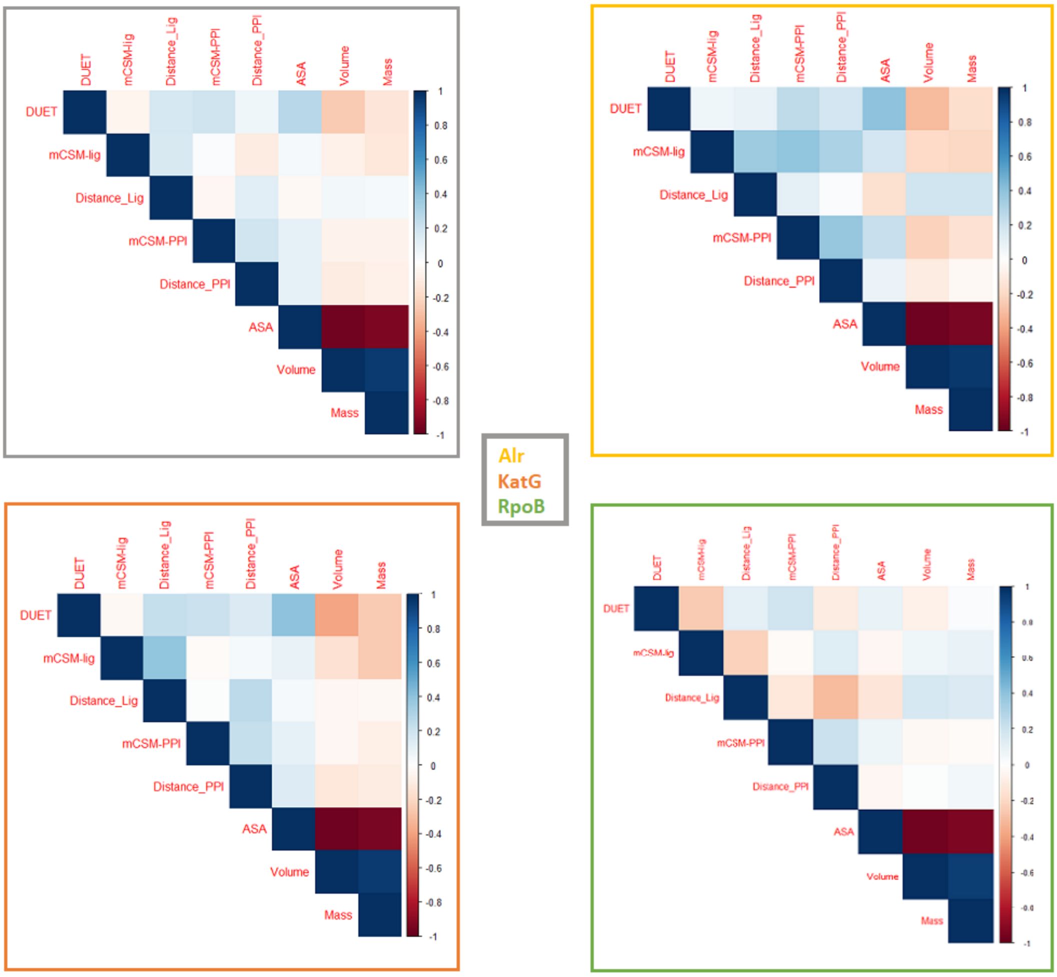

Supplementary Figure 3: Correlations between biophysical (DUET, mCSM-lig, mCSM-PPI) and physicochemical (Accessible Surface Area, Volume, Mass) calculated for collectively (grey) and separately: Alr (yellow), KatG (orange) and RpoB (green). RpoB presented with the lowest associations between ligand and protein-affinities to their respective distances (ρ=-0.23; 0.21), due to its larger structure. Upon decreasing protein complexity in KatG and Alr, higher associations between ligand affinity and distance to ligand (ρ=0.39; 0.35) were observed. A higher association between protein-protein affinity and its respective distance was observed for Alr (ρ=0.38) than for KatG (ρ=0.24), as the latter is a larger homodimer complex. Protein stability was more associated with physicochemical measures ASA and Volume in the smaller proteins KatG (ρ=0.40; -0.40) and Alr (ρ=0.40; -0.31). This was expected since smaller proteins are less tolerable to physical changes than larger ones.

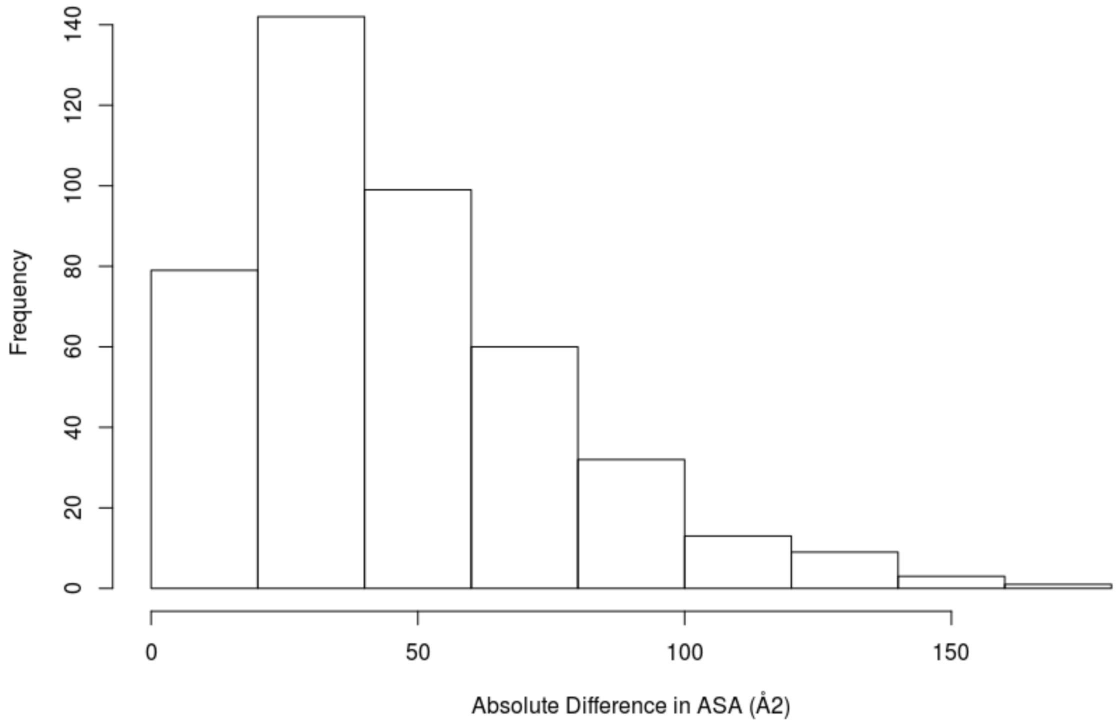

Supplementary Figure 4: Histogram showing the distribution of modulus Accessible Surface Area (ASA) changes upon mutations within alr, katG and rpoB, calculated using values available at: http://cib.cf.ocha.ac.jp/bitool/ASA/
